# Deep Neural Networks for Detection and Location of Microseismic Events and Velocity Model Inversion from Microseismic Data Acquired by Distributed Acoustic Sensing Array

**DOI:** 10.3390/s21196627

**Published:** 2021-10-05

**Authors:** Daniel Wamriew, Roman Pevzner, Evgenii Maltsev, Dimitri Pissarenko

**Affiliations:** 1 Skolkovo Institute of Science and Technology, Bolshoy Boulevard 30, 121205 Moscow, Russia; E.Maltsev@skoltech.ru (E.M.); D.Pissarenko@skoltech.ru (D.P.); 2 Department of Exploration Geophysics, Curtin University, 26 Dick Perry Avenue, Kensington, WA 6151, Australia; R.Pevzner@curtin.edu.au; 3 Total Energies, Research & Development, Lesnaya 7, 125047 Moscow, Russia

**Keywords:** distributed acoustic sensors, microseismic monitoring, neural networks

## Abstract

Fiber-optic cables have recently gained popularity for use as Distributed Acoustic Sensing (DAS) arrays for borehole microseismic monitoring due to their physical robustness as well as high spatial and temporal resolutions. As a result, the sensors record large amounts of data, making it very difficult to process in real-/semi-real-time using the conventional processing routines. We present a novel approach, based on deep learning, for handling the large amounts of DAS data in real-/semi-real-time. The proposed neural network was trained on synthetic microseismic data contaminated with real-ambient noise from field data and was validated using field DAS microseismic data obtained from a hydraulic fracturing operation. The results indicate that the trained network is capable of detecting and locating microseismic events from DAS data and simultaneously update the velocity model to a high degree of precision. The mean absolute errors in the event locations and the velocity model parameters are 2.04, 0.72, 2.76, 4.19 and 0.97 percent for distance (*x*), depth (*z*), P-wave velocity, S-wave velocity and density, respectively. In addition to automation and computational efficiency, deep learning reduces human expert data handling during processing, thus preserving data integrity leading to more accurate and reproducible results.

## 1. Introduction

Fiber-optic cables are increasingly being used as Distributed Acoustic Sensors (DAS) for downhole microseismic monitoring due to their high spatial and temporal resolutions as well as large sensing distances [1] as compared to conventional geophones, which enables them to provide detailed images of the subsurface structure necessary for detection and location of microseismic events as well as velocity model estimation. In addition, DAS continues to be attractive for microseismic monitoring due to its high level of resistance to electromagnetic interference, chemical resistance and good concealment [1].

DAS technology measures the strain or strain-rate along a fiber-optic cable through Rayleigh backscattering of laser pulse [2]. During its operation, an interrogator sends a laser pulse along the fiber-optic cable and records the backscattered light. For each channel, the phase difference between backscattered light within a gauge length is calculated to give the signal. An encounter with a seismic wave causes changes in strain/strain-rate, consequently leading to changes in the recorded signal [2,3]. Due to the short channel (receiver) spacing, DAS is capable of recording unaliased seismic wavelengths as short as a few meters in length when using conventional acquisition parameters. Amongst the many benefits of using fiber-optic cables for downhole microseismic monitoring is that, provided the cable is installed in the well, the system will be able to provide continuous, dense downhole recording while causing no interference with any other activities taking place in or near the well. Because of the adequate fiber coupling provided by this installation, the signal-to-noise ratio is quite high (SNR). Limitations of DAS include lack of broadside sensitivity, as discussed in Section 4.1, and gauge-length effects for small gauge-lengths.

A number of successful DAS applications have been implemented in both land-based and maritime environments, utilizing existing telecommunication fibers. DAS has been implemented in recording earthquakes [4,5,6,7,8,9,10], reconstructing subsurface structures [11,12,13,14,15,16], identifying fault zones [17,18], monitoring traffic [19,20], analyzing oceanic microseisms and tides [21], and recording thunderstorms [22], among other applications. Dedicated downhole deployments, on the other hand, have significantly higher SNR and take full advantage of the sampling resolution and density that DAS provides [23,24,25] by targeting shorter seismic wavelengths. The borehole deployment of sensors also overcomes the near-surface complexity and dissipative nature, which is a hindrance to the analysis of signals recorded at or near the surface [20].

The feasibility of application of DAS for passive microseismic monitoring was first implemented in 2013, and it was validated using collocated conventional geophones [26]. Since then, a lot of improvements have been made on the technology. Because the majority of microseismic monitoring applications are in unconventional reservoirs, fiber-optic cables are frequently used in deviated wells that are aimed at the target reservoir. Traditional detection algorithms were applied to DAS in the early stages, but they were largely ineffective. According to [27], only 31 DAS events were discovered when monitoring hydraulic stimulation, compared to 785 events on the traditional geophones. In addition, [28] quantifies DAS-based events detection to be a mere 10 percent of the geophone-based events. Furthermore, [29] demonstrates that DAS is only capable of detecting events of greater magnitude. On the other hand, the waveform characteristics of microseismic events recorded on DAS were interesting, displaying modes of transformation as well as reflections and scattering. Despite these limitations of event detection capabilities, several attempts have been made to locate the events [28,30]. However, while the advantages of DAS-based location—which primarily consist in positioning an event along the fiber axis—became apparent, the symmetry problem that arises from recording on a single fiber severely curtailed the ability to extract unambiguous event locations from recorded waveforms.

A beamforming approach for event detection and location without azimuthal information was demonstrated by [31] using a single vertical fiber. The detection capabilities of DAS were approximately 30% of those of traditional geophones, suggesting a considerable improvement as compared to the use of conventional detection approaches [32]. After trace-by-trace picking, [33] demonstrates that DAS recordings can be used for travel-time minimization in unconventional reservoirs where horizontal DAS fibers are deployed. This is based on a known velocity structure and is demonstrated in [34]. It is possible to generate reasonable uncertainty in spite of cylindrical symmetry by making several assumptions based on production logs. Arrivals in the deviated and vertical regions of the well can be detected for particular events, allowing many previously degenerate planes to be resolved. In addition, [34] employs deviating well recordings to estimate event location without the necessity for individual channel selection. Instead, they use the DAS records to measure numerous geometrical characteristics and localize events using a constant background model. In terms of observed events, they find that downhole DAS surpasses a surface recording array, which is routinely employed for microseismic monitoring [35], by nearly an order of magnitude.

Machine learning approaches have also been tried and proven to work rather well for inversion of DAS data [36,37]. These approaches, however, largely depend on the quality and size of training datasets and require a significant amount of effort to generate. If trained, neural networks can be shown to generalize easily across diverse sites and offer a lot of potential for event detection [38]. In addition, they can overcome the lower SNR of individual channels by using spatiotemporal patterns across hundreds or thousands of channels at the same time.

Despite the huge progress in development of techniques and routines for detection, location of microseismic events and velocity model inversion, the present routines are largely stand-alone in that they perform only one or two tasks at a time, i.e., either detection and location of the events or velocity model inversion and not all the three. There is, therefore, a need for a technique capable of handling all three tasks of detection, location and velocity model inversion simultaneously in near real-time in order to save time and fast track the decision-making process in the field.

In this study, we demonstrate the feasibility of application of deep learning approach to detect and locate microseismic events and simultaneously estimate the velocity model from DAS-acquired data. Unlike previous studies that use classification approach to detect the events, here, we adopt a regression-based approach in order to perform the three tasks of detection, location and velocity model inversion concurrently. We train the neural network using synthetic DAS data and validate it using both synthetic and field DAS microseismic data.

## 2. Materials and Methods

### 2.1. Field Microseismic Data

We use publicly available field data from phase 2C hydraulic fracture stimulation of the FORGE Research Site near Milford, Utah, USA [39,40]. Three vertical wells were used in the project, for stimulation and monitoring purposes. The monitoring well is 1000 m deep and is located 400 m southeast of the treatment well. A fiber-optic cable was installed in this well, and hydraulic fracture stimulation was conducted in the treatment well. The cable was connected to Silixa iDAS v3 interrogator, which natively measures strain-rate. In addition, data was also acquired using 3C geophones [41].

**Figure 1:**
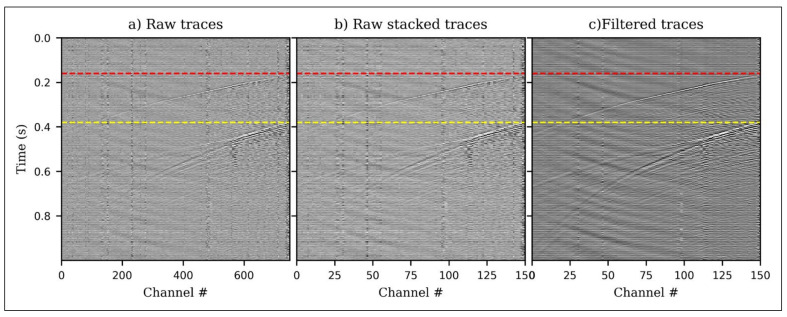
Sample detected event (**a**) raw DAS traces before stacking (**b**) raw traces after stacking (**c**) same traces after 300 Hz minimum phase low-pass and 2D spatial filtering. P arrival (red) and S arrival (yellow).

The iDAS had a gauge length of 10 m and a channel spacing of 1 m along the cable. Data was recorded continuously at a sampling frequency of 2000 Hz throughout the injection period of ~11 days in April–May, 2019. Forty microseismic events were detected and located with moment magnitudes, *M_w_*, in the range −1.653 to −0.519 [41]. For detection of microseismic events, every five traces were stacked to boost the signal-to-noise ratio (SNR) and reduce the data volume. For each stacked trace, the SNR at each time step was computed. This is accomplished by measuring the root mean square (RMS) amplitude 24 ms before and 6 ms after each time sample using sliding windows. The ratio of the RMS values before and after a particular period represents the SNR at that point in time. It will be greatest at the start of an event’s arrival, when the signal is located in the after window and the background noise is located in the before window. A 300 Hz minimum phase low-pass and 2D median spatial filter were applied to attenuate the noise and remove the common-mode noise, respectively. Because DAS only measures single component strain (or strain-rate), the events were constrained to a 2D vertical grid. Figure 1 shows sample traces from the field data.

### 2.2. Training Data

Sixty thousand (60,000) synthetic microseismic events were generated and used in this study to train and optimize the neural network. In generating the synthetic data, we considered 1D anisotropic VTI models with known boundary depths because such models represent the vast majority of geological structures encountered in microseismic monitoring. Strong anisotropy is chosen so that the network, trained on such models, will be able to generalize to lower levels of anisotropy. Six hundred such models were randomly generated with a varying number of layers between 3 and 12. Table 1 summarizes the range of velocity model parameters.

Microseismic events were randomly sampled within a 2-D vertical grid of dimensions 700 m × 900 m. The treatment and the monitoring wells were set 400 m apart, as it were, in the FORGE project. One hundred and fifty receivers with a spacing of 5 m were straddled in the monitoring well from a depth of 1050 m down to 1795 m. For each velocity model, 100 microseismic events were randomly generated within the grid, giving 60,000 events for the 600 models. The focal mechanisms of the events were associated to shear faulting and the corresponding strike, dip and rake angles randomly generated. The events were primarily double couple with some deviatoric components owing to the anisotropy in the velocity models, and they had moment magnitudes, *M_w_*, within the range −2.0 ≤ *M_w_* ≤ 0.

**Figure 2:**
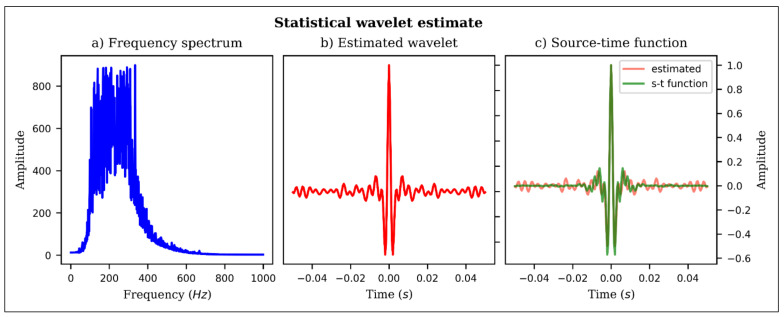
Statistical wavelet estimate from field data, (**a**) mean frequency spectrum of field signal, (**b**) estimated wavelet from field data, (**c**) source time function used in generating synthetic data.

Raytracing was performed to calculate travel times and ray amplitudes of the point sources. Only the vertical channel was considered for particle displacement computation. A statistical estimate of the wavelet was first performed on the field data (Figure 2) to establish the appropriate source time function for the synthetic traces. Consequently, an Ormsby wavelet [43] with the four defining frequencies randomly sampled in the intervals [50–100] Hz, [150–200] Hz, [300–350] Hz and [400–450] Hz, respectively, was loaded onto each source, and displacement seismograms computed. The data was recorded at a 0.5 ms sampling interval for a duration of 1 s and the particle displacements converted to strain-rate. Conversion of vertical particle displacement ($u_{z}$) to strain ($e_{zz}$) along the fiber is straightforward and can be achieved by the following relation [24]:

(1) $$E_{\mathrm{zz}}=\frac{\partial u_{z}}{\partial z}=\frac{\partial u_{z}}{\partial t}\frac{\partial t}{\partial z}=\frac{\partial u_{z}}{\partial t}p_{z}$$

where $p_{z}$ denotes the z-component of the particular phase’s slowness vector. An additional time derivative is performed to obtain the strain-rate because iDAS records strain-rate.

**Figure 3:**
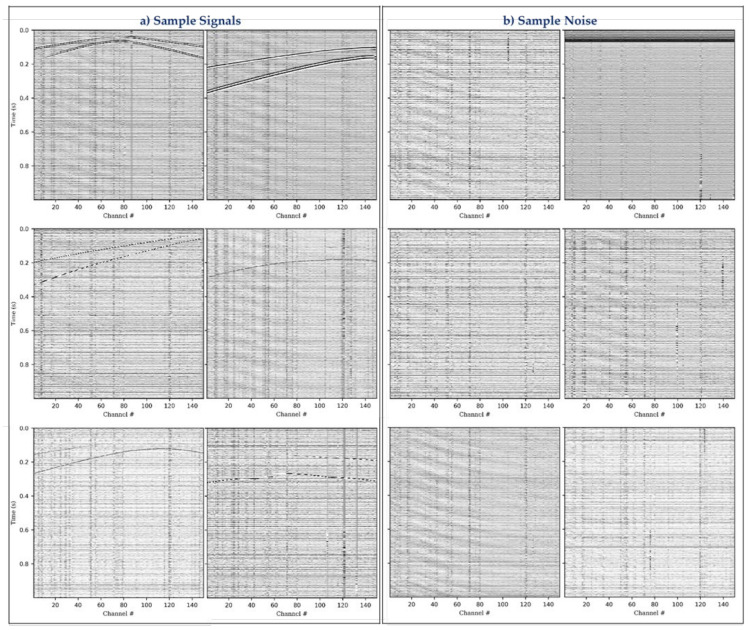
Sample synthetic data (**a**) DAS microseismic events contaminated with ambient noise from field data, (**b**) sample DAS ambient noise extracted from field data.

The seismograms were then contaminated with real-ambient noise from the DAS field records and in addition to the 60,000 events, further 10,000 noise seismograms from the field data were added to the training dataset. The noise was also stacked together and their amplitude normalized to ensure that the amplitudes are comparable before contamination. Figure 3 shows samples of the synthetic events signal and ambient noise.

One sample of the dataset is comprised of a stack of 1-C seismograms from a single event with its corresponding labels comprised of the location coordinates (*x* and *z*) and the velocity model parameters *v_p0_, v_s0_* and *ρ*. For regression purpose, all of the labels for noise were set to zeroes.

### 2.3. Convolutional Neural Network (CNN)

CNNs operate by sliding small filters across the input data matrix and therefore are faster to train [44]. The convolutional layer in a 2D CNN network takes as input three-dimensional matrix and combines it with a set of learnable filters. Each filter is typically small in width and height, compared to the input matrix, but covers the full depth of the input data. During convolution, the filter slides across the width and height of input data, performing pointwise scalar multiplication to produce a two-dimensional activated filter response commonly referred to as activation map. The number of activation maps thus matches the number of filters used in a particular layer. The maps are then stacked along the depth dimension of the block to form the input matrix for the next layer. The output of each point is dependent only upon the height and width of the input matrix because each neuron is initialized from its receptive field in the previous layer. This reduces the number of free parameters, allowing CNNs to handle large amounts of input data.

**Figure 4:**
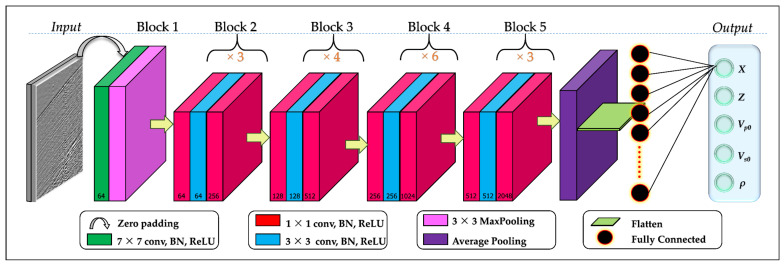
Deep convolutional neural network architecture used in the study. Green, blue and red cuboids represent multi-channel feature maps with number of channels shown at the bottom of the cuboids. The input dimensions of each layer is given in Table 2.

We used a 50-layer deep residual network commonly referred to as ResNet50 [45] to perform the task of inversion of the microseismic data recorded by DAS. This deep neural network overcomes the problem of vanishing gradient as the residual links speed up the network convergence. The network is comprised of 49 convolutional layers and a single fully-connected (FC) layer. The convolutional layers are split in five blocks of 1, 9, 12, 18, 9 and 1 layer(s), respectively, from first to last, with varying kernel sizes and strides. For instance, the single convolutional layer in the first block is comprised of 64 kernels of sizes 7 × 7 and stride of 2 while the first three layers of the second block comprise of 64, 64 and 256 kernels of sizes 1 × 1, 3 × 3 and 1 × 1, respectively, and strides of 2. Table 2 gives a summary of sizes of outputs and convolutional kernels of the network while Figure 4 is a visual presentation of the network architecture. Each convolutional layer was activated using the ReLu activation functions due to its computational efficiency. Maximum and average pooling layers were applied after the first and last convolutional layers, respectively.

The following adjustments were made to ResNet50 in order to accomplish the task at hand: after the last convolution layer, a fully connected layer comprised of 256 nodes was added, followed by the final regression layer comprised of 5 neurons to match the expected output of the microseismic event location and the velocity model parameters. The network architecture is shown in Figure 4.

#### Theory and Implementation of CNN

To accomplish the task of inversion of DAS microseismic data using CNN, the network must be capable of mapping the raw DAS acquired waveforms from data domain to model domain. Deep neural networks achieve this by extracting outstanding features from the waveform data and linking them to the desired output as expressed in Equation (2):

(2) $$\mathrm{Net}(D;\;\theta )\;\to \;\tilde{m}$$

where D is the raw DAS microseismic data and θ represent the network parameters while $\tilde{m}$ is the vector of predictions comprising of event location, *z*, and the velocity model parameters *v_p0_, v_s0_* and *ρ* at the event location.

**Figure 5:**
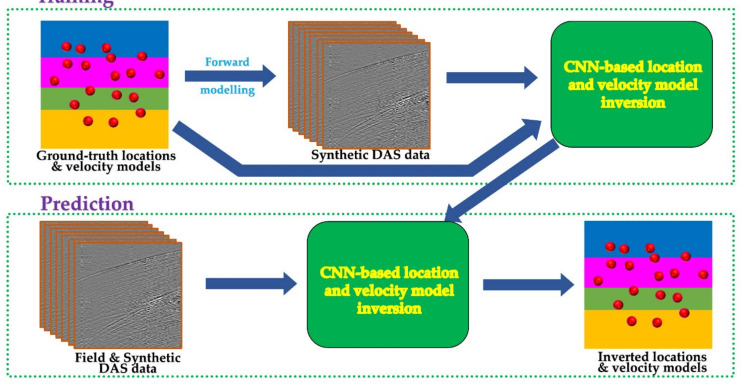
Flow chart depicting CNN-based microseismic events location and velocity model update network. The main processes of forward modeling, training and prediction are highlighted and linked with the arrows. Red dots represent microseismic events.

The supervised learning model consists of two phases: training and validation phase—during which the network learns to associate key features in the input data to the target output; and the testing phase—where the test dataset is passed into the network to obtain predictions. Figure 5 illustrates the flow in the phases.

The network learns to associate key features in the input data to the desired output by solving the optimization problem:

(3) $$\hat{\theta }=\arg\min_{\theta }\frac{1}{N}\overset{N}{\underset{i=1}{\sum }}L\left(m_{i},{\tilde{m}}_{i})\right),$$

where θ denotes the entire network parameters, and L is the mean squared error loss function which evaluates the difference between the ground-truth values $m_{i}$ and the predicted values ${\tilde{m}}_{i}$.

In our case, training the network was accomplished using the Adam algorithm [46], which uses estimates of the first and second moments of the gradients to compute individual adaptive learning rates for different parameters. Because of the large amount of training data and the limited amount of computer memory available, it is not possible to compute the gradient on the entire range of data. As a way around this problem, we loaded data into the network in mini-batches of size h, which was a subset of the entire training dataset in order to evaluate the difference between the predicted and ground-truth values, $L_{h}$, in each iteration. As a result, we adopted the following formulation for the optimization task:

(4) $$\hat{\theta }=\arg\min_{\theta }\frac{1}{h}L_{h}=\arg\min_{\theta }\frac{1}{h}\overset{h}{\underset{i=1}{\sum }}{\left\|m_{i}-\mathrm{Net}(D_{i};\theta )\right\|}_{2}^{2}.$$

Here the velocity models and the true locations are provided during the training and validation phases but are invisible to the network during testing. The parameters of the network were updated using the Adam algorithm [46]:

(5) $$\theta_{t+1}=\theta_{t}-\alpha \frac{{\hat{M}}_{t}}{\sqrt{\mu_{t}}+\epsilon },$$

where M and μ are the corrected bias estimators for the first and second moments respectively, while α is the learning rate (step size) and ϵ is chosen so small to avoid division by zero. This approximation is simple to implement, efficient in computation, and well-suited for problems involving large amounts of data and parameters that must be learned, such as in our case.

### 2.4. Training and Validation of the Network

The dataset was split as follows: 5000 samples comprising of 50 velocity models were reserved for testing while the remaining 65,000 was split in the ration 7:3 for training and validation respectively. The test dataset was only fed into the network to obtain the predictions after the training was complete. The seismograms were converted to grey scale images of pixel dimensions 256 × 256 before input into the network. The data was input in minibatches of size 32 after prior tests indicated that smaller or larger minibatch sizes did not improve the network’s performance. Two precautionary measures were taken to avoid overfitting: first, we tracked the model’s performance on the validation dataset after each epoch and only saved its weights if its performance improved on the validation dataset. Second, we used a validation dataset comprised of 30% of the entire dataset, randomly sampled, to evaluate the network’s performance after each epoch of training. The neural network was built and trained using the open-source Python package Keras, which was run on a TensorFlow backend. We used a GeForce GTX 1080 Ti GPU for training and the model trained for 135 epochs before reaching convergence. The training took 7.2 h.

## 3. Results

### 3.1. Synthetic Data Analysis

**Figure 6:**
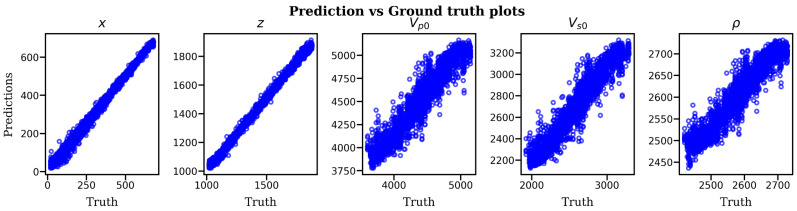
Scatter plots of predictions versus ground-truth values of event depth location and velocity model parameters *v_p0_, v_s0_* and *ρ*. The plots show strong positive correlation.

The test dataset comprised of 5000 DAS microseismic events from 50 distinct velocity models was used to evaluate the performance of the trained network. As a first step in evaluating the model’s performance, we input the entire test dataset and plot the scatter diagram (Figure 6) of predictions vs. ground-truth values to determine the correlation between the predictions and the actual values. The scatter plots for both location and velocity model estimates show strong positive correlation between the predicted and the ground-truth values, indicating that deep learning is capable of inverting the raw DAS microseismic data for the detection, location and velocity model inversion.

**Figure 7:**
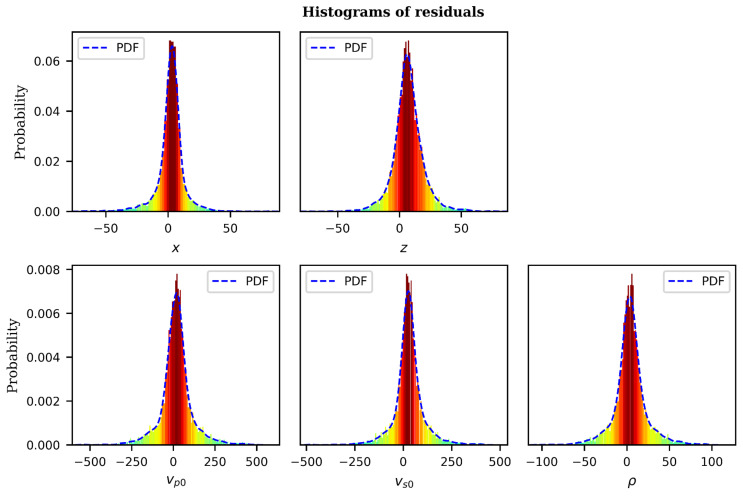
Residual diagnostic histograms for trained CNN model. The blue envelope represents the probability density function. The velocity model parameters of each histogram are indicated at the bottom center of each plot.

To examine the goodness-of-fit of the trained model, we performed residual diagnostics. Two rules of thumb are verified; i.e., for a good model, the residuals should randomly deviate from zero, and secondly, the residuals should be close to zero themselves. Figure 7 displays the histograms of residuals for each parameter under study and an envelope of probability density function.

The distribution of the residuals for the trained model, as seen on Figure 7, follow a normal distribution for all five parameters under study. The mean of the residuals is very close to zero for all the parameters, which indicates compliance with statistical assumptions that residuals have zero-mean and constant variance. Having verified the suitability of the model, we proceed to evaluate its output for each of the five parameters.

**Figure 8:**
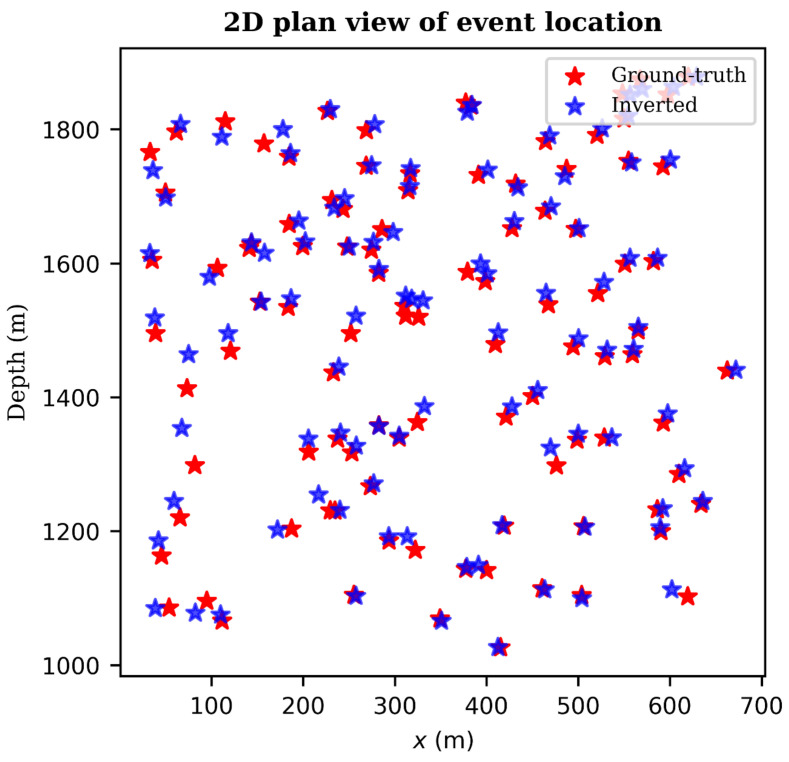
2D plan view projection of the inverted DAS microseismic depth locations (blue stars) versus ground-truth locations (red stars). Only 100 events from one velocity model are displayed.

In order to visualize the relative locations of the predicted and ground-truth events, we plot a 2D plan view projection of the event locations as shown in Figure 8. For clarity, we plotted only 100 events from a single velocity model. From Figure 8, it is clear that the predicted event locations (blue stars) almost perfectly match the ground-truth locations (red stars) with very minimal deviations in some cases.

**Figure 9:**
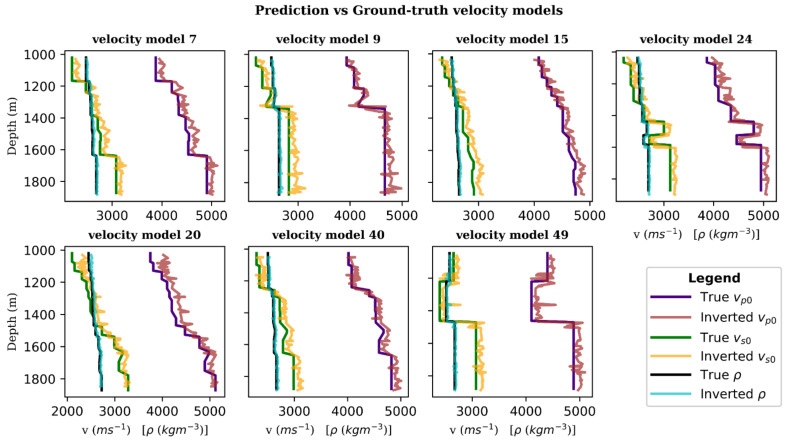
1D velocity model profiles for prediction versus ground-truth values. Blue, green and black represent ground-truth *v_p0_, v_s0_* and *ρ* values respectively, while red, orange and cyan the corresponding inverted values.

Further, we compare the velocity model parameters from the predictions against the ground-truth values in the velocity versus depth profiles displayed in Figure 9 to quantify the accuracy of the velocity model predictions. Evidently, the predicted velocity models, to a large extent, match the patterns of the ground-truth models.

**Figure 10:**
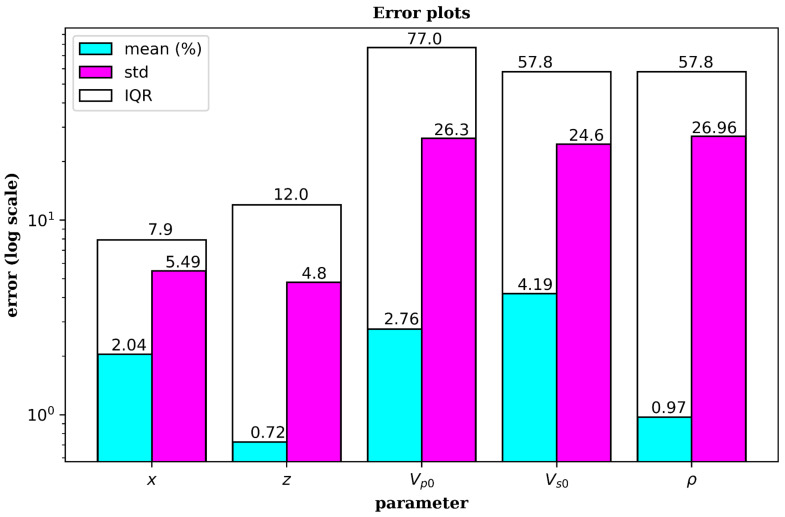
Error bar graph for the location and velocity model parameters inverted by deep learning. The vertical scale is logarithmic while the parameters are shown on the horizontal axis with their respective values at the top of each bar. Cyan, purple and white bars represent the mean absolute error, standard deviation and the interquartile ranges, respectively.

To further validate the capability of the proposed approach, we performed statistical analysis of the prediction results for all the five parameters under study. A summary of the results is presented on Figure 10. The mean absolute errors in the inversion are 2.04, 0.72, 2.76, 4.19 and 0.97 percent for *x, z, v_p0_, v_s0_* and *ρ*, respectively, while the corresponding standard deviations are 5.49, 4.80, 26.30, 24.60 and 26.96. We observe that while the errors in the predicted velocity models are minimal, the corresponding standard deviations are somewhat high compared to that of inverted locations. This can be attributed to two reasons. First, the number of velocity models used in the inversion are significantly smaller than the number of microseismic events, meaning we have more information on the latter. Second, while the number of microseismic events was maintained constant in all the velocity models, the number of layers in a velocity models varied between 3 and 12. Hence, the neural network may have not adequately learned the properties of the velocity models in comparison to the event locations. The results can be improved by increasing the number of velocity models in the inversion and a maintaining constant number of layers throughout the experiments.

### 3.2. Field Data Analysis

In this section, we test the limits and potential of the deep-learning approach with field data from FORGE project, discussed in Section 2.1 above. The microseismic database consists of 15 s long SEG-Y data files collected over a period of ~11 days, with events of different magnitudes. We chose a three-hour subset of the data, cycles 7 and 8 of stages 27 and 28, confirmed to contain 30 events [41]. We split the data to one-second lengths using a sliding window to give a time sample of 2000 time steps for use in the pre-trained neural network. We then applied 300 Hz minimum low pass filter followed by a 2D median filter to attenuate the noise and remove the common-mode noise, respectively. The entire test dataset thus contained 10,800 samples which were amplitude-normalized and then converted to grey scale images of pixel size 256 × 256. No further processing was done.

**Figure 11:**
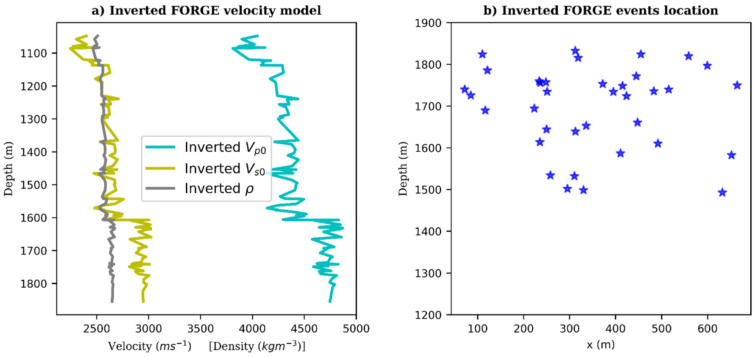
Inversion results: (**a**) Velocity model of FORGE estimated by the neural network. Cyan, yellow and grey curves are the raw patterns output of *v_p0_, v_s0_* and *ρ* by the network, (**b**) Events locations.

For testing the pre-trained CNN model, we input the entire test dataset into the network and obtain predictions of event locations and velocity model parameters *v_p0_, v_s0_* and *ρ*. Figure 11 shows plots of the inverted depth locations of the events and the estimated velocity model.

In addition to the 30 previously confirmed events, the neural network was able to detect and locate six more low magnitude events that had not been previously reported. Determination of the events moment magnitudes is, however, beyond the scope of this study.

## 4. Discussion

Both DAS and deep learning are promising new technologies in microseismic monitoring. A combination of both could be a game changer. DAS has, in the recent times, become a favorite alternative for acquisition of microseismic data due to its spatial and temporal resolution and physical robustness. However, the large volumes of data originating from DAS sensors make it very difficult to process in real-/semi-real-time using the conventional routines. This is where deep learning comes in. While there are plenty of standard tools for seismic event detection, location and velocity model inversion, the deep learning approach combines all three stages and, as such, saves the time. In the foregoing sections, we have attempted to demonstrate the feasibility of application of deep learning approach for inversion of microseismic data acquired by DAS.

Results for both synthetic and field data from the FORGE enhanced geothermal project near Milford, Utah depict the potential of deep learning approach for inversion of DAS acquired data. We were able to detect and locate microseismic events from DAS records to a high degree of accuracy and approximate the velocity model (Figure 11). The tasks can be performed in real-time, in the case of hydraulic fracturing operation, or in semi-real-time, in case of passive seismic monitoring. The proposed approach could help both petroleum and mining engineers fast-track field decision-making processes and assist in production optimization. However, it must be validated with long-term monitoring and data from different formations.

In generating the training dataset, we used ray tracing; however, it is possible to use other methods such as reflectivity or full waveform inversion. Ray tracing was chosen due its versatility and numerical efficiency. When performed in a smoothly varying layered media, ray tracing is capable of delivering reliable approximate solutions with sufficient levels of accuracy. Its primary limitation is that, because it is an approximate solution to the wave equation, it is only practically functional in smoothly varying medium and might produce incorrect results or even fail in singular regions [47]. As mentioned in forward modeling, we used 1D layered anisotropic velocity models but more accurate results can be achieved using a 3D model and iterative methods.

### 4.1. Limitations of DAS

The sensitivity of DAS is dependent on the angle of incidence of seismic energy with respect to the orientation of the fiber optic cable due to well-known properties underlying the system’s operation. Generally, DAS is most sensitive to seismic signals incident along the axial direction of the sensing optical fiber. On the contrary, the system is less sensitive to those signals that are incident perpendicular to the axial direction of the sensing fibers, that is, broadside signals.

**Figure 12:**
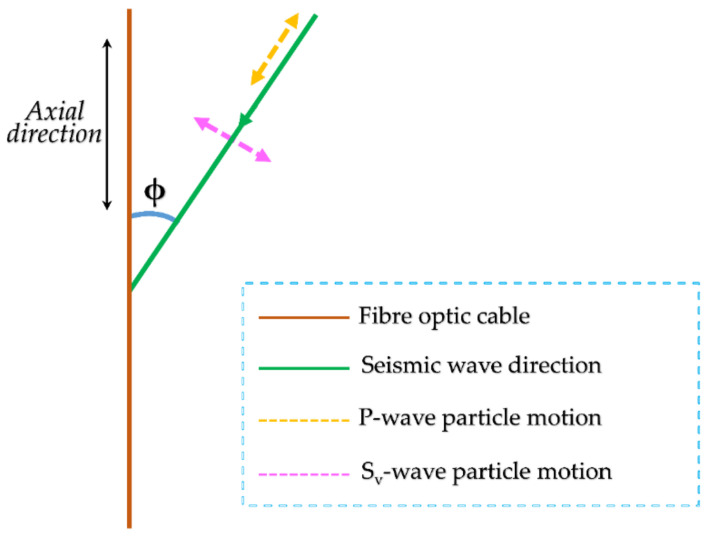
Directional sensitivity of DAS to body waves. DAS is most sensitive to the component of particle motion that is in the axial direction.

It is worth noting that this directionality is linked to the phase of the seismic wave that arrives at a specific incidence angle. If the angle of incidence, ϕ, (Figure 12) is defined as 0 degrees for arrivals propagating along the fiber’s axial direction and 90 degrees for arrivals propagating perpendicular to the optical fiber, the sensitivity to P-wave arrivals is greatest for angles of incidence approaching 0 and least for angles approaching 90 degrees. On the contrary, because S-waves propagate perpendicular to directions of motion, their sensitivity increases as the incidence angle approaches 90 degrees and diminishes for angles closer to 0.

### 4.2. Limitations of Deep Learning

Due to their large architectures, deep learning models require large volumes of data for training. While availability of data is not a challenge in microseismic/passive seismic monitoring using DAS, the large volumes of data make training of the models extremely computational expensive. Moreover, a model trained in one situation will requre retraining to be applied to a different situation. Once trained, however, the models are computationally efficient.

In addition, despite their excellent performance on the benchmark dataset, deep learning models may fail on a field dataset if there are significant differences between the field data and the training data. For field application of deep learning, it is a major challenge to generate the characteristics and links between data domain and model domain.

### 4.3. Field Implementation

The deep learning approach can be easily implemented in the on-site automated field-data-processing workflow. A field experiment where DAS data acquisition and processing was automated was recently published by Isaenkov et al., [48]. In that study, an array of five deep wells generates ~1.3 Tb of raw data per day, and the data is processed as active time-lapse VSP. The data pre-processing was done in a similar way to the way we prepared the data for analysis in this study.

As explained in Section 3.2 above, the pre-processing involves filtering, downsampling and conversion of gathers to greyscale images. The images are then fed to the pre-trained CNN model for inversion. The neural network outputs the locations of detected events as well as an estimate of the velocity model. The inversion process is very fast; for instance, it took 673 milliseconds on an octa-core CPU to process 1.3 GB of pre-processed data. In the case of hydraulic fracturing, data can be streamed in real-time, thus reducing the input data size and decreasing the processing time.

## 5. Conclusions

The results of this study show that deep neural network models are capable of learning the relationship between microseismic waveform data and the location of the events as well as the velocity model to a high degree of precision. Furthermore, the tasks of updating the velocity model and locating events are carried out concurrently on a single network. The deep-learning approach has numerous advantages that make it more appealing for real/semi-real-time microseismic monitoring. Most importantly, the approach requires minimal data pre-processing because the model is capable of learning and interpreting the properties of recorded waveform data by itself, in order to detect and locate microseismic events and invert velocity models. As a result, the uncertainties associated with data handling and processing by human experts are eliminated. Furthermore, because more data is acquired, the network’s performance can be improved in real-time during training. The model is computationally efficient once it has been trained; for instance, the inversion of the five thousand test dataset only took 673 milliseconds on an octa-core CPU.

Future research should focus on evaluating the neural network model’s performance on more complicated geological formations with various degrees of anisotropy. In order to build innovative neural network designs capable of managing a wide range of complicated geological formations, the idea of combining conventional techniques with specialized deep learning models should be investigated.

## Figures and Tables

**Table 1:** Range of parameters used to generate the velocity models. Thomsen anisotropic parameters [42] of ε = 0.51, γ = 0.36, and δ = 0.25 were taken to be constant throughout the layers.

Parameter	v_p0_ (m/s)	v_s0_ (m/s)	ρ (kg/m^3^)	Depth (m)
Minimum	3830	2193	2466	1000
Maximum	5059	3187	2711	1900

**Table 2:** Dimensions and number of convolutional kernels in the 50-layer residual network (ResNet-50).

Layer Name	Output Size	Number of Layers
Conv1	128×128	7×7, 64, stride 2
Conv2_x	3×3 max pool, stride 2	
	64×64	$\left[\begin{array}{c} 1\times 1,64\\ 3\times 3,64\\ 1\times 1,256 \end{array}\right]\times 3$
Conv3_x	32×32	$\left[\begin{array}{c} 1\times 1,128\\ 3\times 3,128\\ 1\times 1,512 \end{array}\right]\times 4$
Conv4_x	16×16	$\left[\begin{array}{c} 1\times 1,256\\ 3\times 3,256\\ 1\times 1,1024 \end{array}\right]\times 6$
Conv5_x	8×8	$\left[\begin{array}{c} 1\times 1,512\\ 3\times 3,512\\ 1\times 1,2048 \end{array}\right]\times 3$
Fully connected	5×1	Average pool, 256, linear
**Total parameters**		24,106,692

## Data Availability

We used both synthetic and field data from Utah Forge hydraulic fracturing project. The real data is publicly available here http://gdr.openei.org/submissions/1207 (accessed on 5 May 2021). All the codes are written in Python and are partly available on Github here: https://github.com/wamriewdan/das_microseismics_inversion (accessed on 12 September 2021). We are happy to release the codes to reproduce all our results on Github on publication of this paper. The corresponding author may be contacted, if need be, concerning access to the synthetic data used in the project.
